# Supplementary material for: Parenterally Administered P24-VP8* Nanoparticle Vaccine Conferred Strong Protection against Rotavirus Diarrhea and Virus Shedding in Gnotobiotic Pigs
Source: Vaccines (Basel). 2019 Nov 6;7(4):177. doi: 10.3390/vaccines7040177 (PMC6963946; doi:10.3390/vaccines7040177)
Supplement: Supplementary file 1 [file vaccines-07-00177-s001.pdf]

vaccines

Supplementary Materials

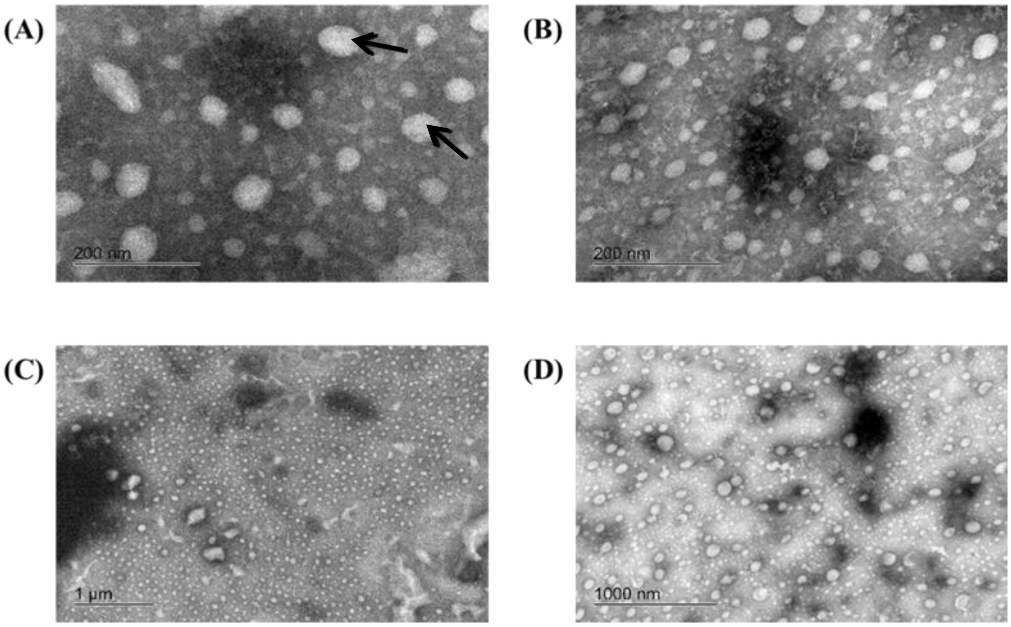

Figure S1. Negative stain TEM images of P24-VP8* particles (indicated by black arrow) taken over two different time points. Panels (**A**) and (**C**) were taken 3 months after vaccine preparation and panels (**B**) and (**D**) were imaged 8 months after vaccine preparation.

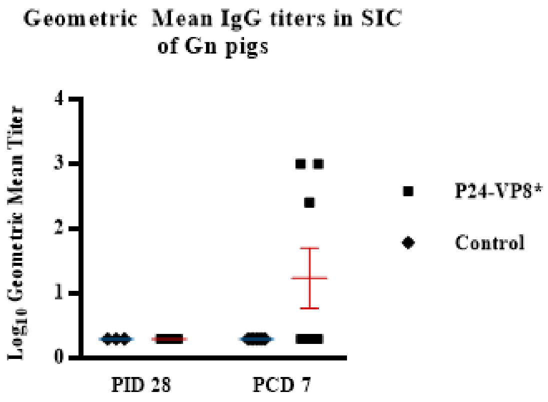

Figure S2. Geometric mean VP8*-specific IgG titers in SIC samples collected upon euthanasia at PID 28 and PCD 7. Negative samples were assigned an arbitrary value of 2 for statistical calculations and graphical illustration. Three out of 8 pigs were observed to be positive for VP8*-specific IgG antibodies. There were no significant differences.
